# Impact of body mass index on surgical outcomes and delay adjuvant treatment in patients undergoing therapeutic mammoplasty for breast cancer

**DOI:** 10.1016/j.jpra.2026.01.021

**Published:** 2026-01-25

**Authors:** Haifa Alotaibi, Romany Mikhael, Ian Nunney, Reginald Salvador, Angeline Embuscado, Sendhil Rajan, Maged Hussien

**Affiliations:** a Breast Surgery Unit, Norfolk and Norwich University Hospital, Norwich, UK; b University of East Anglia, Norwich, UK

**Keywords:** Therapeutic mammoplasty, Breast reduction, Breast conserving surgery, Adjuvant treatment

## Abstract

**Background:**

Therapeutic mammoplasty (TM) is an oncoplastic technique that combine wide excision of cancer with breast reduction, enabling breast conservation in patients with moderate-to-large breasts. The effect of body mass index (BMI) on TM and oncological outcomes remains controversial. This study aims to evaluate the complication rate in high BMI patients undergoing TM and to assess the impact on timing of adjuvant therapy

**Patients and methods:**

Retrospective review of patients underwent TM between January 2014 and January 2024. Data recorded include age, weight, height, BMI, smoking, comorbidities, tumor type and size, lymph node status, neoadjuvant / adjuvant treatment and its timing. Surgery performed by one surgeon. All patients had contralateral reduction at the same session. Type of pedicle, weight of removed breast tissue, and postoperative complications were recorded. Statistical analysis was performed using the appropriate statistical test.

**Results:**

About 73 patients were included in the analysis. Patient’s mean age was 61 years (range: 58–67), Mean BMI 29.1 kg/m² (range: 20–43). The average weight of tissue removed per breast was 653 g (range: 219–948 g). Overall complications rate (32.8 %). Two patients returned to theatre for drainage of hematoma (2.7%) and the remaining minor wound complications were treated as outpatient. About 45 patients (61.6%) had BMI < 30 with 15 patients developed wound complication (33%). 28 patients (38.4%) had BMI ≥ 30 with nine patients developed wound complications (32%) *P* = 0.99.

No significant association between complication rates and all the recorded parameters. There was no significant delay in the initiation of adjuvant therapy (chemotherapy or radiotherapy) between patients who experienced complications (mean waiting time 70 Days) and those who did not (mean waiting time 77 days) *P* = 0.78. One patient developed local recurrence and another patient developed distant metastasis after overall median follow up 53 months (IQ 30–89 months).

**Conclusions:**

TM is a safe technique for breast cancer surgery. According to our experience, patients with high BMI (>30 kg/m^2^) can be managed with TM as this was not associated with increased complications or delay in adjuvant therapy.

## Introduction

Breast-conserving surgery (BCS) has emerged as the preferred treatment for early-stage breast cancer, offering low recurrence rates and survival outcomes comparable to mastectomy.^1^ Some studies even suggest that oncological outcomes following BCS may be superior to those achieved with mastectomy, highlighting its efficacy as a treatment modality.^2^^,^^3^ Despite these advantages, BCS can be technically challenging and may result in suboptimal cosmetic outcomes, which can negatively impact patient satisfaction and overall quality of life.^4^

To address these limitations, oncoplastic breast surgery techniques have been developed, combining oncological safety with aesthetic principles to enable adequate tumor resection without compromising breast appearance. Therapeutic mammoplasty (TM), first described by Clough et al. in 1990 in patients undergoing breast reduction surgery for lower pole cancers, has since evolved into a versatile oncoplastic approach applicable to tumors in any breast quadrant using various vascular pedicle designs.^4^ TM integrates principles of oncologic resection with breast reduction and mastopexy techniques, allowing for wider excision margins while simultaneously reshaping the remaining breast tissue to maintain contour and symmetry. An additional advantage of TM is the reduction of breast volume, which can facilitate postoperative radiotherapy. Several studies have demonstrated that patients with large breasts are at higher risk of developing early and late radiation-induced toxicities of ≥ Grade 2.^5^^,^^6^ Both short- and long-term outcomes of TM have demonstrated favorable oncological and cosmetic results, establishing it as a safe and effective option for patients requiring larger resections or with large ptotic breasts.^7^

Despite these advantages, patient-related factors such as obesity may influence both the aesthetic and clinical outcomes of TM. High body mass index (BMI) is associated with a significantly increased risk of postoperative complications, with obese patients demonstrating nearly 12-fold higher odds of complications following elective breast procedures compared to those of normal weight. While complication rates are elevated across all breast procedures in obese patients, the impact is particularly pronounced in more complex surgeries, such as TM.^8^ Postoperative complications may not only affect immediate recovery but can also delay the initiation of adjuvant treatments. Evidence indicates that delays exceeding 56 days between surgery and radiotherapy, or more than 90 days between surgery and chemotherapy, are associated with increased locoregional recurrence and poorer survival outcomes, respectively.^7^

While the effects of obesity on outcomes following aesthetic breast reduction have been well studied, its impact when similar techniques are applied in the oncologic setting has not been fully explored. Given the rising prevalence of obesity^9^ and the growing use of oncoplastic techniques, understanding the impact of BMI on surgical outcomes and timely administration of adjuvant therapy is critical. The aim of this study is to evaluate the effect of BMI on the rate of postoperative complications and delays in adjuvant treatment in patients undergoing therapeutic mammoplasty.

## Methods

### Study design and setting

This was a retrospective review of all patients electronic documents who underwent TM between January 2014 and January 2024 at Norfolk and Norwich University hospital- United Kingdom. The study was conducted in accordance with the hospital privacy policy for data usage.

### Patient selection and data collection

All patients with breast cancer treated with TM under care of one surgeon during the study period were included. Patients with incomplete medical records or those who underwent procedures other than TM were excluded. Demographic, clinical, and pathological data were extracted from electronic medical records and operative notes. Variables collected included patient age, weight, height, BMI, smoking status, and comorbidities. Tumor-related data included histological type, tumor size, and lymph node status. Treatment-related variables included the receipt of neoadjuvant therapy and the type and timing of adjuvant therapy. All patients had contralateral breast reduction for symmetrization in the same surgery.

Surgical details recorded included the type of pedicle used, weight of excised breast tissue, and perioperative findings. All operations were performed by the same senior breast oncoplastic surgeon, ensuring consistency of technique.

In addition, patient-reported outcomes were assessed using the validated BREAST-Q questionnaire to evaluate quality of life and satisfaction domains. These results will be analyzed and presented in a separate publication.

### Outcomes

The primary outcomes were postoperative complications rate and the interval to initiation of adjuvant therapy. Complications were defined as wound infection, dehiscence, seroma, hematoma, fat necrosis, nipple–areolar complex necrosis, lymphedema, or need for reoperation. For adjuvant treatment, the interval from surgery to initiation of radiotherapy or chemotherapy was recorded. Rather than using predefined cut-offs, the time to adjuvant therapy was compared between patients who developed postoperative complications and those who did not. These intervals were also compared to the average time to adjuvant therapy for breast cancer patients undergoing conventional breast-conserving surgery at our unit during the same period. Long-term data on oncological outcomes, including survival and local recurrence were recorded until December 2025.

### Statistical analysis

Statistical analyses were performed using *SAS version 9.4.* Descriptive statistics for continuous variable having a normal distribution, were summarized using the mean and respective 95% confidence intervals or when a normal distribution could not be assumed the median and respective interquartile range (IQR) were reported. Categorical variables were presented as frequencies and percentages. Associations between BMI and postoperative complications or treatment delays were assessed using chi-square tests for categorical variables, and the Student’s t-test or the Mann–Whitney U test for continuous variables correcting the type 1 error rate for multiple comparisons. For the binary outcome, having a complication, a multiple logistic regression analysis was performed to analyze if there was any association with BMI adjusting for weight reduction, treatment delay and smoking or diabetes. A *P*-value of <0.05 was considered statistically significant.

## Results

### Patient characteristics

A total of 73 patients who underwent TM and contralateral reduction between January 2014 and January 2024 were included in the analysis. The mean age was 61 years (range 58–67), and the mean BMI was 29.1 kg/m² (range 20–43). The average weight of resected breast tissue was 653 g (range 219–948). Table 1. The type of pedicle used were either supero-medical or inferior pedicle for nipple areola complex.

**Table 1:** Patients characteristics.

Characteristic	Complication		Overall
	No.	Yes	
BMI			
Number of patients	49	24	73
Mean (SD)	29.2 (5.8)	29.1 (4.6)	29.1 (5.4)
			*P* = 0.76
Age			
Mean (SD)	61.9 (9.0)	61.6 (10.7)	61.8 (9.5)
			*P* = 0.83
Weight of breast tissue removed (one side) g			
Mean (SD)	636.5 (680.9)	686.1 (559.8)	653.0 (638.2)
			*P* = 0.48
Lymph node status			
Negative (%)	32 (66.7)	13 (59.1)	45 (64.3)
Positive (%)	16 (33.3)	9 (40.9)	25 (35.7)
Total	48	22	70 (three patients had no LN surgery)
			*P* = 0.53
BMI group			
<25	15	2	17
25–34	18	19	37
>35	16	3	19
			*P* = 0.9
Diabetes/Smoking			
Yes	6	5	11
No	43	19	62
Total	49	24	73
			*P* = 0.33

### Postoperative complications

The overall complication rate was 32.8% (n = 24). Two patients (2.7%) required return to theatre for evacuation of hematoma, while all other complications were minor wound-related issues managed on an outpatient basis.

**Figure 1:**
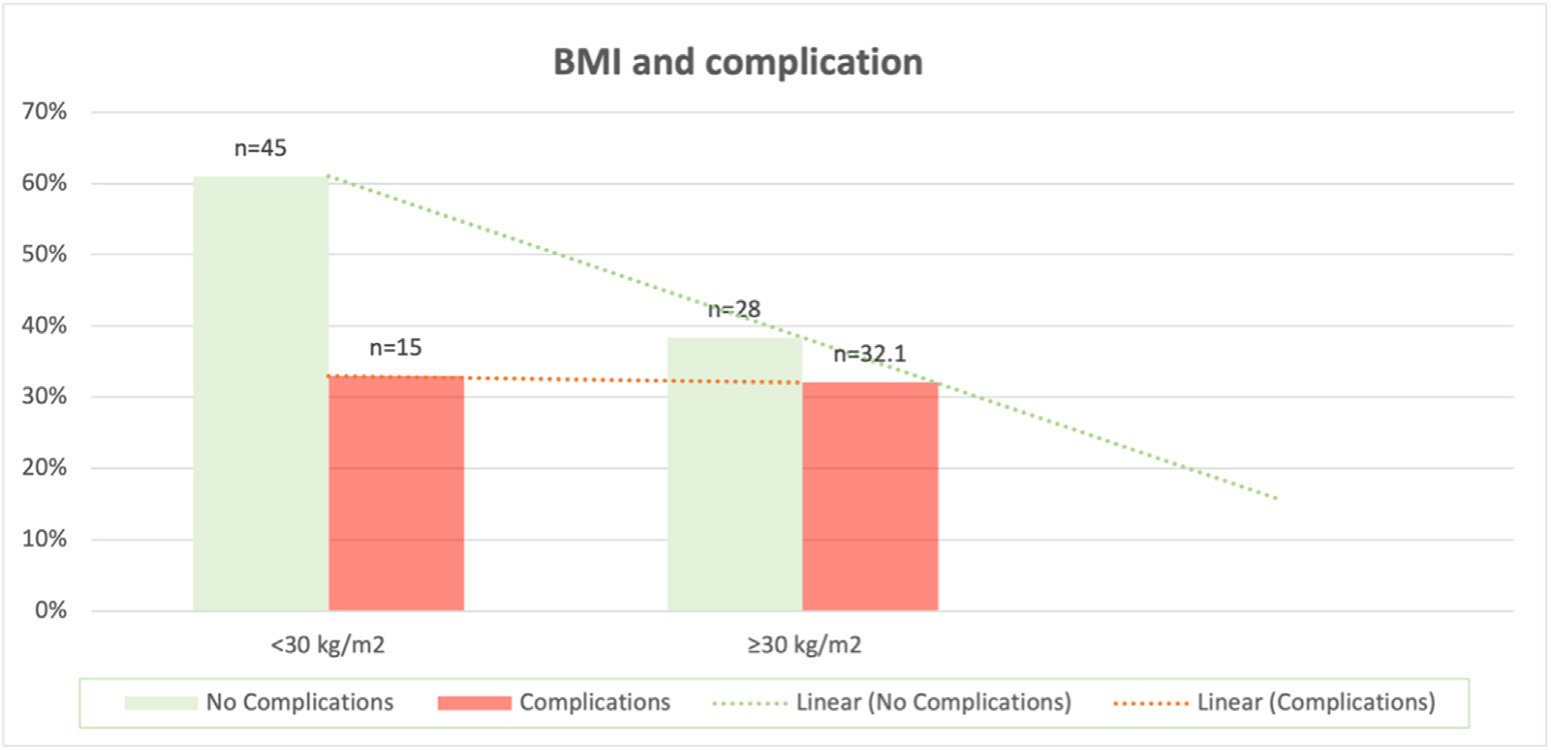
BMI and complication rate.

When stratified by BMI, 45 patients (61.6%) had BMI < 30, of whom 15 (33.0%) developed complications. Twenty-eight patients (38.4%) had BMI ≥ 30, of whom nine (32.1%) developed complications. There was no significant difference in complication rates between the two groups (*P* = 0.99) Figure 1. Among the cohort, only 11 patients were identified as current or former smokers or as having diabetes mellitus. Of these, eight patients (73%) had a BMI between 25 and 34, while only two patients (18%) had a BMI greater than 35. Analysis of the association between these comorbidities and postoperative complications demonstrated no statistically significant difference (*P* = 0.33).

### Subgroup analysis by BMI and complication rates

Subgroup analysis was performed using different BMI categorizations (<25, 25–34, and ≥35) to ensure that the method of BMI classification did not introduce bias. The results were consistent across all categorizations. About 17 patients had BMI <25 with two patients developed wound complication. About 37 patients had BMI 25–34 and 19 patients developed wound complications. About 19 patients had BMI >35 with only three patients had wound complications. A higher rate of complications was observed in the 25–34 BMI group, which may be explained by the fact that the majority of the cohort fell into this category (n = 37 patients).

### Adjuvant therapy timing

**Figure 2:**
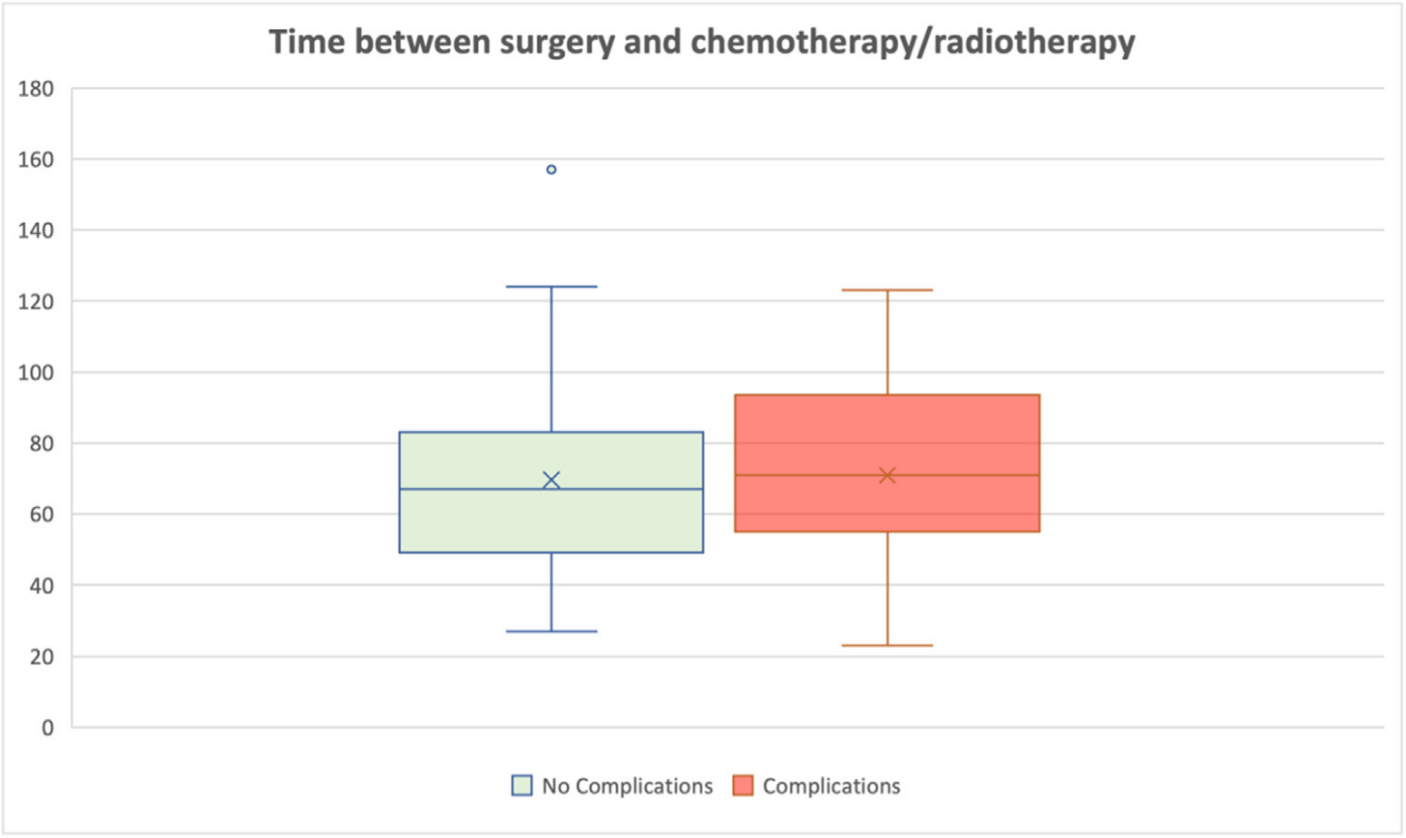
Interval to adjuvant therapy.

The mean time to initiation of adjuvant therapy was 70 days for patients who developed complications and 77 days for those without complications. This difference was not statistically significant (*P* = 0.78). Furthermore, the observed intervals were comparable to the average time to adjuvant therapy for patients undergoing conventional breast-conserving surgery within the unit Figure 2.

### Local recurrence and survival

One patient had her surgery in December 2020 for invasive carcinoma and developed metastasis of mediastinal lymph nodes and bones in July 2025. Patient remains alive on treatment up to the date of preparation of this article. Another patient had her surgery in February 2023 for large area of DCIS and developed recurrent ipsilateral DCIS and Paget’s disease in August 2025 and had completion mastectomy. The median follow up period was 53 months (range 30–89 months).

## Discussion

In our cohort of 73 patients, 38.3% were classified as obese, using a BMI of 30 kg/m² or greater as the cut-off based on the World Health Organization (WHO) classification. This relatively high proportion of obese patients reflects the increasing prevalence of obesity in the general population, with recent UK data estimating that 26.9% of adult women were obese in 2023–2024.^10^ Using a BMI of 30 kg/m² as the threshold, there was no significant difference in complication rates between obese and non-obese patients (33% vs. 32%). However, when patients were further subclassified into BMI < 25, 25–34, and ≥ 35, a higher rate of complications was observed in the BMI 25–34 group. This may be attributed to the larger number of patients within this subgroup (n = 37 patients), which could have influenced the statistical distribution.

Several studies have explored the association between obesity and postoperative outcomes in breast surgery, with varying conclusions. Obesity has been reported to increase the odds of postoperative complications by nearly 12-fold following elective breast surgery. The complication rate is consistently higher in obese compared to normal-weight patients across all breast procedures. However, the effect of obesity appears most pronounced in more complex surgeries, where baseline complication rates are already elevated.^11^

For instance, a large study involving 2403 obese and 5597 non-obese women undergoing breast operations reported overall complication rates of 18.3% and 2.2%, respectively, including infection, delayed wound healing, seroma, hematoma, and implant removal.^11^ In contrast, Zubowski et al. reviewed 267 bilateral breast reduction cases and found no significant difference in major systemic complications across BMI categories.^12^ Similarly, Setälä et al. found no correlation between obesity and the overall risk of postoperative complications among 273 patients who underwent bilateral breast reduction.^13^ Likewise, Roehl et al. performed a retrospective analysis of 179 reduction mammaplasty cases and found no statistically significant difference in complication rates based on BMI; however, there was a clear trend toward increasing complication rates with higher BMI values.^14^ Our findings are consistent with the latter studies, suggesting that obesity alone may not significantly increase the risk of overall complications following reduction or therapeutic mammaplasty.

Wound healing issues and wound dehiscence were the most frequently observed complications in our cohort, consistent with findings reported in previous studies.^15^, ^16^, ^17^ All patients who developed complications were managed in the outpatient setting, with only two patients required reoperation for hematoma evacuation.

Available literature is divided on whether resection weight is correlated with a higher incidence of surgical complications.^12^^,^^17^, ^18^, ^19^ In our study, larger resection volumes were not associated with a higher risk of complications (*P* = 0.48).

Other factors, such as diabetes mellitus (DM), which is more prevalent in individuals with higher BMI, may also influence wound complication rates. Smoking has likewise been reported to increase the risk of postoperative complications.^20^^,^^21^ In our analysis, we examined the association between DM and smoking with complication rates; however, this relationship was not statistically significant (*P* = 0.33). It is worth noting that only 11 patients in our cohort had DM or were smokers, which may have limited the statistical power of this analysis.

The primary concern regarding wound complications following therapeutic mammoplasty is the potential delay in initiating adjuvant therapy, which has been reported to negatively impact oncological outcomes.^22^^,^^23^ In our cohort, the mean time to commence any adjuvant treatment was 75 days (*P* = 0.78), reflecting the institutional average, with no significant difference between patients who experienced complications and those who did not. This delay is likely attributable to prolonged pathology turnaround times. This interval is comparable to that reported by R. Rampal et al. for patients with complications, where similar delays did not adversely affect local recurrence rates (LRR), disease-free survival (DFS), or overall survival (OS).^24^ However, our interval was longer than the 54-day median reported in the TeaM study, which included patients from 48 centers across the UK and two centers in Italy.^25^

This study has several limitations that should be acknowledged. Its retrospective design and relatively small cohort, particularly with few patients having diabetes mellitus or who were smokers, may have influenced the statistical analysis of these subgroups. Data on neoadjuvant therapy were not collected, although this factor could potentially contribute to wound complication rates.

Long-term oncological outcomes were assessed. One patient (1.3%) had local recurrence and another patient (1.3%) developed distant metastasis during the study period with median follow up 53 (range 30–89) months. Future prospective studies with larger cohorts would be valuable to further investigate these factors and validate our findings. Few studies have evaluated survival and local recurrence after therapeutic mammoplasty. Emiroglu 2017, evaluated survival and local recurrence in 82 patients who had therapeutic mammoplasty.^26^ Local recurrence was 8.7% and survival 82.2% at median follow up 121 months.^26^

Deigni 2020, compared complication rate and delay in adjuvant treatment in patients who had symmetrizing contralateral reduction at the same surgery or as a delayed procedure. They concluded that contralateral mastopexy/breast reduction for symmetry can be performed at the time of breast-conserving surgery without significantly increasing the risk of complications or delay to adjuvant radiation therapy.^27^ In our study, all patients had their symmetrizing contralateral surgery at the same time.

Fitzpatrick 2020, published a review article on the incidence of occult breast carcinoma found in the breast reduction surgery specimens and reported that this incidence is higher if women had history of breast cancer.^28^ In our study, there was no occult breast cancer detected in any of the contralateral breast reduction specimens, which may reflect improvement of preoperative imaging assessment.

## Declaration of competing interest

None declared.
